# Application of Intraoperative Neuromonitoring (IONM) of the Recurrent Laryngeal Nerve during Esophagectomy: A Systematic Review and Meta-Analysis

**DOI:** 10.3390/jcm12020565

**Published:** 2023-01-10

**Authors:** Boyang Chen, Tianbao Yang, Wu Wang, Weifeng Tang, Jinbiao Xie, Mingqiang Kang

**Affiliations:** 1 Department of Cardiothoracic Surgery, The Affiliated Hospital of Putian University, Putian 351100, China; 2 Department of Cardiothoracic Surgery, Nanjing Drum Tower Hospital, The Affiliated Hospital of Nanjing University Medical School, Nanjing 210008, China; 3 Department of Thoracic Surgery, Fujian Medical University Union Hospital, Fuzhou 350001, China

**Keywords:** esophagectomy, neuromonitoring, meta-analysis, recurrent laryngeal nerve, IONM

## Abstract

Background: recurrent laryngeal nerve palsy (RLNP) is a common and severe complication of esophagectomy in esophageal cancer (EC). Several studies explored the application of intraoperative neuromonitoring (IONM) in esophagectomy to prevent RLNP. The purpose of this study was to conduct a systematic review and meta-analysis to evaluate the value of IONM in esophagectomy for EC. Methods: an electronic of the literature using Google Scholar, PubMed, Embase, and Web of Science (data up to October 2022) was conducted and screened to compare IONM-assisted and conventional non-IONM-assisted esophagectomy. RLNP, the number of mediastinal lymph nodes (LN) dissected, aspiration, pneumonia, chylothorax, anastomotic leakage, the number of total LN dissected, postoperative hospital stay and total operation time were evaluated using Review Manager 5.4.1. Result: ten studies were ultimately included, with a total of 949 patients from one randomized controlled trial and nine retrospective case–control studies in the meta-analysis. The present study demonstrated that IONM reduced the incidence of RLNP(Odds Ratio (OR) 0.37, 95% Confidence Interval (CI) 0.26–0.52) and pneumonia (OR 0.58, 95%CI 0.41–0.82) and was associated with more mediastinal LN dissected (Weighted Mean Difference (WMD) 4.75, 95%CI 3.02–6.48) and total mediastinal LN dissected (WMD 5.47, 95%CI 0.39–10.56). In addition, IONM does not increase the incidence of aspiration (OR 0.4, 95%CI 0.07–2.51), chylothorax (OR 0.55, 95%CI 0.17–1.76), and anastomotic leakage (OR 0.78, 95%CI 0.48–1.27) and does not increase the total operative time (WMD −12.33, 95%CI −33.94–9.28) or postoperative hospital stay (WMD −2.07 95%CI −6.61–2.46) after esophagectomy. Conclusion: IONM showed advantages for preventing RLNP and pneumonia and was associated with more mediastinal and total LN dissected in esophagectomy. IONM should be recommended for esophagectomy.

## 1. Introduction

The recurrent laryngeal nerve (RLN) lymph node (LN) is the most frequent site of LN metastasis in esophageal cancer (EC) and an important prognostic factor for EC [1,2,3]. Routinely, the lymphadenectomy of RLN LN is one of the critical steps in the esophagectomy of EC and is very important for accurate staging and the improvement of long-term survival [4,5].

However, despite the oncologic benefits, RLN LN dissection is a highly invasive procedure. Recurrent laryngeal nerve palsy (RLNP) may be caused by thermal injury, stretching, compression, or vascular compromise to RLN due to its deep anatomical location and poor visualization, with a reported incidence of 9–22% [6,7,8]. In Asia, the incidence of RLNP can be up to 59%, mainly attributed to extensive three-field lymph node dissection [9]. Patients with RLNP often suffer from hoarseness, aspiration, pneumonia, and dyspnea, which can be even more fatal in those who undergo esophagectomy [10]. Due to the risk of adverse outcomes described, some surgeons have even had to compromise between these complications and long-term survival [11]. 

Intraoperative neuromonitoring (IONM) detects the stimulation of the RLN by sensing the electromyographic signal of vocal muscle movement, which helps to identify the RLN and properly reduces medically induced injuries [12]. IONM has gained wide acceptance and has become an adjunct to the gold standard of visually identifying RLN and reducing RLN injury in thyroid and parathyroid surgery [13]. In 2001, Hemmerling et al. first reported the application of IONM in esophagectomy [14]. In the following decades, several studies have further explored the application of IONM in esophagectomy; however, the results are controversial. Therefore, the purpose of this study was to conduct a systematic review and meta-analysis to evaluate the value of IONM in esophagectomy for EC.

## 2. Materials and Methods

### 2.1. Literature Search

A systematic review and data synthesis were conducted according to a pre-defined, registered (PROSPERO, CRD42022354833) and published protocol following PRISMA 2020 statement [15]. The systematic literature searches of Google Scholar, PubMed, Embase, and Web of Science databases were performed using terms comprising “esophagectomy”, “esophagus”, “esophageal”, “intraoperative”, “nerve monitoring”, “neuromonitoring”, “neural monitoring”, “IONM”, and “nerve detector” with the Boolean operators “AND” or “OR” (data up to 31 October 2022). In the first stage, all the literature was screened by title and abstract. In the second stage, potentially eligible examples of the literature were reviewed in their full text to determine eligibility for inclusion-exclusion criteria. The literature search and review were performed independently and duplicated by two reviewers. References from the eligible literature were further checked for additional suitable studies. Any disagreement was resolved through discussion. The reasons for exclusion were recorded.

### 2.2. Inclusion-Exclusion Criteria

Inclusion criteria included: (1) Studies comparing IONM-assisted and conventional non-IONM-assisted esophagectomy. (2) Patients with esophageal malignancy who underwent esophagectomy combined with mediastinal lymph node dissection. Exclusion criteria included: (1) Conference abstracts, review articles, and case reports. (2) Non-comparative studies.

### 2.3. Outcomes of Interest

The primary outcome measures were postoperative complications, including RLNP and the number of mediastinal LN resected. Secondary outcome measures were the total LN resected, total operation time, postoperative hospital stay (POHS), and other postoperative complications such as pneumonia, hoarseness, aspiration, chylothorax, and anastomotic leakage.

### 2.4. Data Extraction and Assessment of Methodological Quality

Data regarding participant characteristics included the study design, author, the number of participants, surgical details, operation time, POHS, and postoperative complications. For some outcomes, data were recorded as medians with quartiles or ranges, and an online conversion tool www.math.hkbu.edu.hk/~tongt/papers/median2mean.html (accessed on 25 December 2022) was used to convert the data to means and standard deviations (SD) [16,17]. The Cochrane risk-of-bias tool was used to assess RCTs and the Newcastle–Ottawa quality assessment scale (NOS) for non-RCTs.

### 2.5. Statistical Analysis

Statistical analysis and plotting were performed using Review Manager 5.4.1 (Cochrane Collaboration, Oxford, UK). Dichotomous variables from the relevant studies were extracted, and the corresponding pooled odds ratios (ORs) and 95% confidence intervals (CIs) were calculated by the Mantel–Haenszel method. For continuous variables, the weighted mean differences (WMDs) with 95% CIs were calculated by the inverse variance method. A random effects model was applied to make the statistical results more reliable and appropriately conservative [18]. Higgins’ I^2^ statistic was used to determine the percentage of total variations across the studies due to heterogeneity. Publication biases were assessed when the number of included studies was greater than 10. The publication bias was qualitatively estimated using funnel plots. The Egger test was conducted to evaluate the publication bias when the funnel plot was visually inspected as asymmetry, and the “trim and fill” test was further accessed if the publication bias was detected [19]. 

### 2.6. Ethics Statement

This study is a meta-analysis based on publicly available data and does not include human subjects or require Institutional Review Board (IRB) approval.

## 3. Result

### 3.1. Identification of Studies and Characteristics

**Figure 1:**
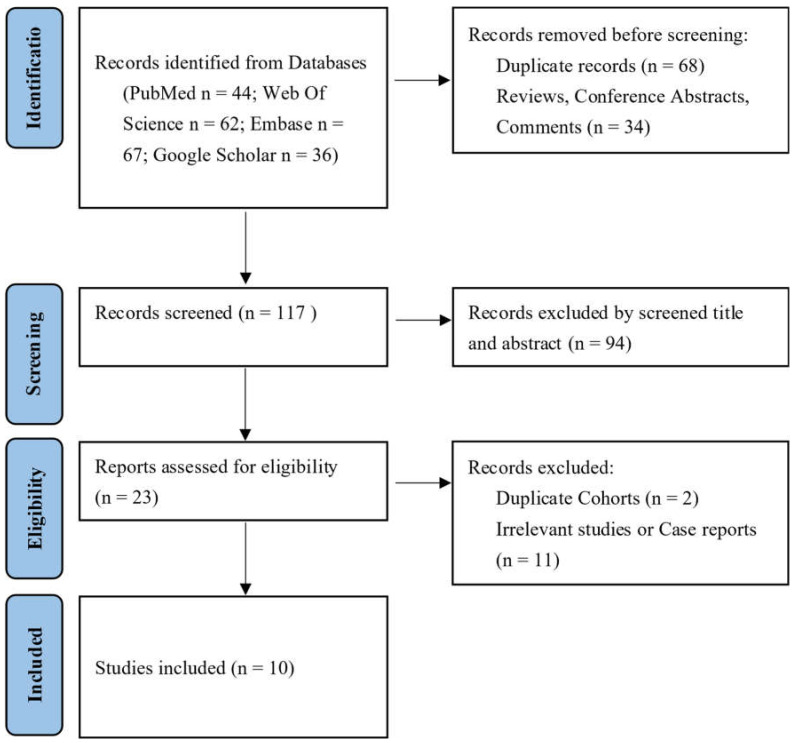
Flow chart of the literature search according to preferred reporting items for systematic reviews and meta-analyses statement.

A total of 106 references that were considered potentially relevant were screened from PubMed, EMBASE, and Web of Science. Through the screening of titles and abstracts, 39 papers were identified for full-text review, and 10 studies that met the inclusion-exclusion criteria were ultimately included in this study. The literature screening process is shown in Figure 1. This meta-analysis contained nine retrospective case–control studies and one randomized control trial [20,21,22,23,24,25,26,27,28,29]. A total of 949 patients were included in the study, of which 508 patients were treated with IONM, and 441 patients were the non-IONM control. The characteristics of the included studies are summarized in Table 1. The literature quality assessment of each study included in the analysis is summarized. (Supplementary Table S1).

### 3.2. RLNP

**Figure 2:**
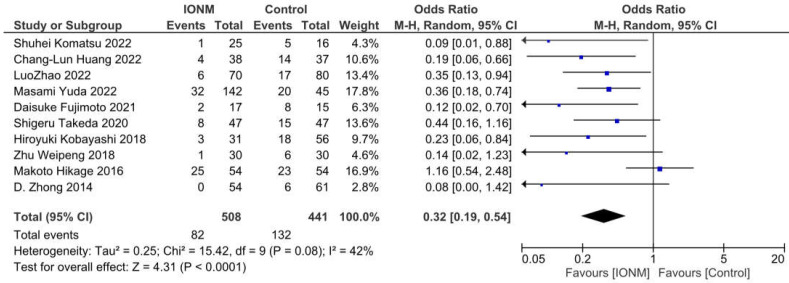
Forest plot of IONM for RLNP. Abbreviations: RLNP—recurrent laryngeal nerve palsy, IONM—intraoperative neuromonitoring.

RLNP was reported in all 10 studies and occurred in a total of 214 among 949 patients. The Forest plot is shown in Figure 2. The Pooled analysis shows that IONM is associated with a reduced incidence of RLNP after esophagectomy (OR = 0.32, 95%CI (0.19, 0.54), *p* = 0.08; I^2^ = 42%). A sensitivity analysis was further performed as I^2^ was close to 50%. The results of the pooled analysis remained robust after omitting any of the included studies. (Supplementary Table S2). The funnel plot showed asymmetry visually, and Egger’s test showed a significant publication bias. (Egger’s test, *p* = 0.02) The “Trim and Fill” test was further accessed, and no trimming was performed, which showed that the result was robust.

### 3.3. Number of Mediastinal LN Dissected

**Figure 3:**
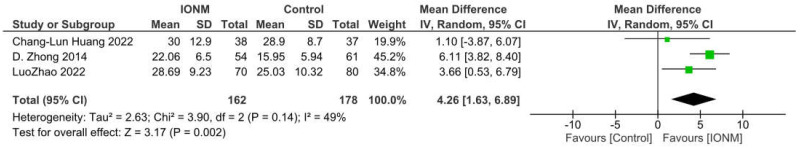
Forest plot of IONM for number of mediastinal LN dissected. Abbreviations: IONM—intraoperative neuromonitoring.

The number of mediastinal LN dissected was reported in three studies among 340 patients. The Forest plot is shown in Figure 3. The Pooled analysis showed that IONM was associated with significantly more mediastinal LN dissected after esophagectomy (WMD = 4.26, 95%CI (1.63, 6.89), *p* = 0.14; I^2^ = 49%). The sensitivity analysis showed that the results of the pooled analysis were not statistically significantly different after omitting Luo Zhao’s study. (Supplementary Table S3).

### 3.4. Aspiration

**Figure 4:**
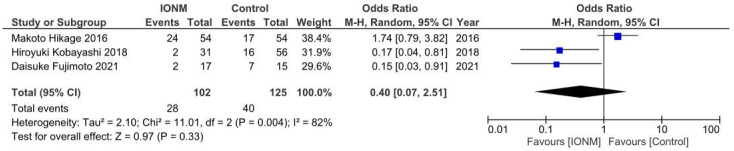
Forest plot of IONM for aspiration. Abbreviations: IONM—intraoperative neuromonitoring.

Aspiration was reported in three studies and occurred in a total of 68 among 227 patients. The Forest plot is shown in Figure 4. The Pooled analysis showed no statistical difference between IONM and the incidence of aspiration (OR = 0.4, 95%CI (0.07, 2.51) 0.004, I^2^ = 82%). Sensitivity analysis showed a decreased incidence of aspiration after omitting Makoto Hikage’s study. (Supplementary Table S4).

### 3.5. Pneumonia

**Figure 5:**
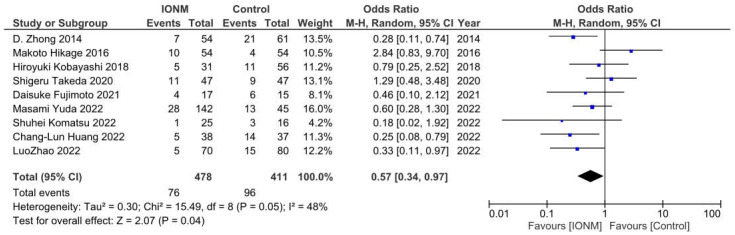
Forest plot of IONM for pneumonia. Abbreviations: IONM—intraoperative neuromonitoring.

Pneumonia was reported in nine studies and occurred in a total of 172 among 889 patients. The Forest plot is shown in Figure 5. The Pooled analysis showed that IONM is associated with a reduced incidence of pneumonia (OR = 0.57, 95%CI (0.34, 0.97), *p* = 0.05, I^2^ = 48%). The sensitivity analysis showed that the results remained robust after omitting any of the included studies. (Supplementary Table S5).

### 3.6. Chylothorax

**Figure 6:**
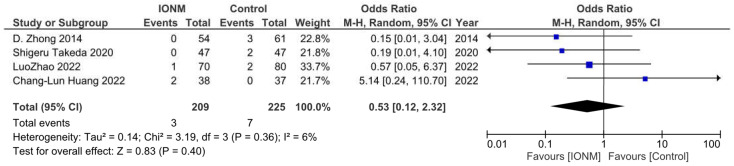
Forest plot of IONM for chylothorax. Abbreviations: IONM—intraoperative neuromonitoring.

Chylothorax was reported in four studies and occurred in a total of 10 among 434 patients. The Forest plot is shown in Figure 6. The Pooled analysis showed no statistical difference between IONM and the incidence of chylothorax (OR= 0.53, 95%CI (0.12, 2.32), *p* = 0.36, I^2^ = 6%). The sensitivity analysis showed that the results remained robust after omitting any of the included studies. (Supplementary Table S6).

### 3.7. Anastomotic Leakage

**Figure 7:**
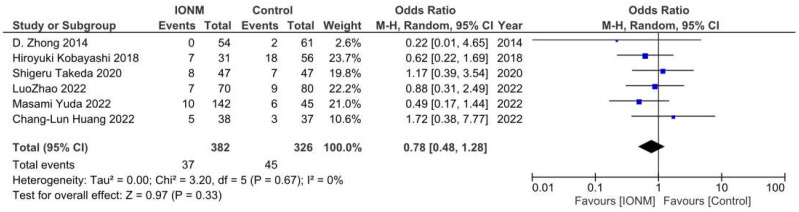
Forest plot of IONM for anastomotic leakage. Abbreviations: IONM—intraoperative neuromonitoring.

Anastomotic leakage was reported in six studies and occurred in a total of 82 among 708 patients. The Forest plot is shown in Figure 7. The Pooled analysis showed no statistical difference between IONM and the incidence of anastomotic leakage (OR = 0.78, 95%CI (0.48, 1.28), *p* = 0.67, I^2^ = 0%). The sensitivity analysis showed that the results remained robust after omitting any of the included studies. (Supplementary Table S7).

### 3.8. Number of Total LN Dissected

**Figure 8:**
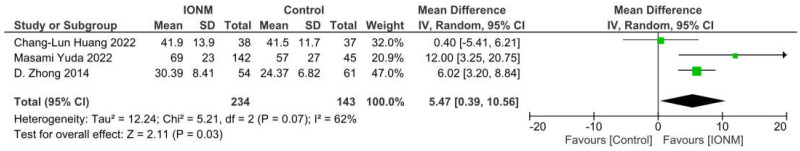
Forest plot of IONM for total LN dissected. Abbreviations: IONM—intraoperative neuromonitoring.

The number of total LN dissected was reported in three studies among 377 patients. The Forest plot is shown in Figure 8. The Pooled analysis showed that IONM was associated with a significantly higher total LN dissected after esophagectomy (WMD = 5.47 95%CI (0.39, 10.56) *p* = 0.03; I^2^ = 62%). The sensitivity analysis showed that the results of the pooled analysis were not statistically or significantly different after omitting Masami Yuda and D. Zhong’s study. (Supplementary Table S8).

### 3.9. Postoperative Hospital Stay (POHS)

**Figure 9:**
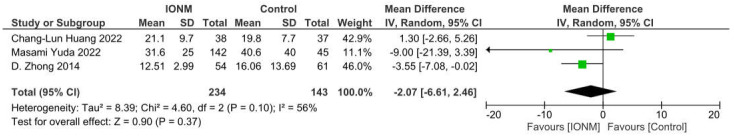
Forest plot of IONM for POHS. Abbreviations: IONM—intraoperative neuromonitoring, POHS—postoperative hospital stay.

POHS was reported in three studies among 568 patients. The Forest plot is shown in Figure 9. The Pooled analysis showed no statistical difference between IONM and POSH after esophagectomy (WMD = −2.07 95%CI (−6.61, 2.46) *p* = 0.10; I^2^ = 56%). The sensitivity analysis showed a decreased POHS after omitting Chang-Lun Huang’s study. (Supplementary Table S9).

### 3.10. Total Operation Time

**Figure 10:**
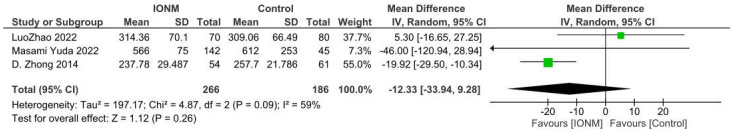
Forest plot of IONM for total operation time. Abbreviations: IONM—intraoperative neuromonitoring.

The total operation time was reported in three studies among 452 patients. The Forest plot is shown in Figure 10. The Pooled analysis showed no significant difference in the total operative time between the IONM and controls (WMD= −12.33, 95%CI (−33.94, 9.28) *p* = 0.09; I^2^ = 59%). The sensitivity analysis showed a decreased total operation time after omitting LuoZhao’s study. (Supplementary Table S10).

## 4. Discussion

The electrophysiological monitoring of RLN can be traced back to as early as 1966 by Shedd and Durham [30]. The commercially available endotracheal tube with surface electrodes for IONM was first reported in 1996 and soon gained wide recognition in thyroid and head and neck surgery [31]. According to a survey, more than half of the otolaryngologists and general surgeons have applied IONM in surgery [32,33]. A recent meta-analysis demonstrated that IONM significantly reduced the total injury of RLN in thyroidectomy. (RR = 0.68, 95%CI (0.55–0.83)) (12). Currently, the application of IONM in thyroid surgery has been recommended by various clinical guidelines [13,34,35]. 

RLN LN is also the most metastatic LN of thoracic esophageal squamous carcinoma. Although complete lymph node dissection significantly improved long-term survival for esophageal patients, the rise in comorbidities annoys surgeons, and it was reported that esophagectomy has a more than four times higher incidence of injury to RLN than thyroid surgery [36,37]. In 2001, Hemmerling first reported the application of IONM in a patient of esophageal cancer with extensive RLN LN involvement [14]. In 2010, Hans Gelpke reported the first prospective study of IONM for lobectomy and esophagectomy and confirmed its feasibility [38]. D. Zhong published the first comparative study of IONM in esophagectomy in 2014, which showed that IONM reduced the incidence of RLNP and pneumonia and improved the mediastinal LN resection [29]. In 2021, Xinxin Wang published the first meta-analysis of IONM in esophagectomy, including five retrospective studies, and suggested that IONM was associated with lower rates of RLNP and hoarseness and more effective lymphadenectomy [39]. 

Our meta-analysis included additional studies, and all were published within 10 years. These studies were all from East Asia, and the pathological type was mainly squamous cell carcinoma. Most studies used the McKeown procedure, except for the Sweet procedure (left thoracotomy) by D. Zhong and transhiatal esophagectomy by Shuhei Komatsu [20,29]. The Medtronic system was applied for IONM in all included studies. The laryngeal electromyography was monitored using a Medtronic NIM Contact Reinforced EMG Endotracheal tube, and the automatic periodic stimulation system used a Medtronic NIM-Response 3.0 with the APS Electrode system.

Our meta-analysis showed that IONM is associated with a reduced incidence of RLNP and more mediastinal and total LNs dissected. A variety of factors may be attributed to the advantages of IONM. Firstly, RLN can be easily identified by stimulation probes and thus avoid injury. Especially in patients who received neoadjuvant therapy, the fibrotic tissue after tumor regression in the upper mediastinum and poorly defined margins due to tissue edema is difficult to distinguish from RLN visually. IONM not only helps to identify RLN but also enhances the surgeon’s confidence [23]. Secondly, inappropriate traction on nerves is another mechanism that causes RLN injury. When the real-time alarm is triggered by inappropriate traction, the nerve injury can be transient if the surgeon releases the traction immediately [40]. Thirdly, the recently proposed concept of mesenteric excision suggests that the mesoesophagus in the upper mediastinum has potential lymphatic drainage and micro-metastases and should be removed radically [41]. The application of IONM fits with the mesenteric excision theory. RLN is wrapped in the mesoesophagus, and IONM helps predict the location of RLN before visual contact, which makes the dissection using an energy device adjacent to the RLN much safer and allows for the en-bloc resect of the mesoesophagus and LNs [26,42]. 

Although the Funnel plot and Egger’s test showed a significant publication bias, the “Trim and Fill” test proved that our results were still robust. IONM showed an obvious superiority in almost all studies. As Shuhei Komatsu stated in his article, “Because clinical benefit was obvious, it was not possible to have a group of patients without CNM(IONM) for comparison” [20]. IONM may be so stable and effective that there is a lack of negative results. Our study also revealed that IONM reduced the incidence of pneumonia. Postoperative pneumonia is the most common complication after esophagectomy, with an incidence of up to 60% [43]. Several recent studies showed that RLNP is associated with pneumonia, and multivariate analysis showed that RLNP is the primary independent risk factor for pneumonia. (RR = 2.3–6.2) [9,44]. Our study supports the previous studies that IONM can reduce pneumonia by preventing RLNP. Although inconsistent with a previous meta-analysis, our results are more reliable due to the larger number of studies included and insignificant heterogeneity [39]. A study suggests that RLNP is an independent prognostic factor for pneumonia and POHS [9]. In addition, we found that IONM does not increase the operative time and the risk of other common complications, which reflected the feasibility of IONM.

The present study has several limitations. First, all the included studies were not randomized control trials; therefore, bias was inevitable. Second, most of the studies are single-centered with relatively small sample sizes, and some studies used historical control groups. This limited the statistical power. Thirdly, this study lacks an analysis of the impact of IONM on postoperative survival. Finally, due to the heterogeneity in this study, the results are interpreted with caution.

## 5. Conclusions

Our meta-analysis demonstrated that the application of IONM during esophagectomy reduced the incidence of RLNP and pneumonia and was associated with more mediastinal and total LN dissected. In addition, IONM does not increase the incidence of aspiration, chylothorax, and anastomotic leakage and does not increase the total operative time or postoperative hospital stays. IONM should be recommended for esophagectomy. Large RCTs are still needed to provide more evidence in future studies.

## Figures and Tables

**Table 1:** Characteristics of included studies.

Author	Year of Publication	Study Period	Region	Study Design	Group	SEX (Male/Female)	Age (Mean ± SD)	Tumor Location (Upper/Middle/Lower)	Pathology (SCC/AC/Other)	Neoadjuvant Therapy (NO/CT/CRT)	N Stage (N0/N1/N2/N3)	Stage (0/I/II/III/IV)
Shuhei Komatsu [20]	2022	2014–2019	Japan	RCS	IONM	19/6	72 (53–81) ^a^	3/13/9	38/3/0	19/6 ^b^	11/6/7/1	0/8/6/10/1
					Control	10/6	62.99 ± 8.81	5/45/20		11/5 ^b^	8/4/1/3	0/8/2/3/3
Chang-Lun Huang [21]	2022	2015–2020	ROC	RCS	IONM	34/4	67 (47–80) ^a^	21/38/25	33/4/1	23/15 ^b^	19/8/11/0	N/A
					Control	34/3	67 (53–77) ^a^	5/10/26	34/2/1	17/20 ^b^	17/13/7/0	
LuoZhao [22]	2022	2016–2020	China	RCS	IONM	59/11	68 (41–84) ^a^	8/25/21	N/A	45/22/3	N/A	0/25/18/22/5
					Control	70/10	72 (50–85) ^a^	3/7/6		57/21/2		0/27/16/34/3
Masami Yuda [23]	2022	2011–2018	Japan	RCS	IONM	118/24	61.96 ± 7.12	6/49/25	133/5/4	52/86/4	65/45/21/11	0/36/55/53/9
					Control	77/11	68 (54–81) ^a^	11/20/16	39/3/3	21/24/0	25/8/7/5	0/12/15/16/2
Shigeru Takeda [24]	2020	2009–2018	Japan	RCS	IONM	40/7	67.5 (53–80) ^a^	6/23/27	N/A	21/26 ^b^	N/A	0/17/9/14/7
					Control	41/6	65 (49–80) ^a^	3/29/22		20/27 ^b^		1/18/5/14/9
Daisuke Fujimoto [25]	2021	2015–2018	Japan	RCS	IONM	13/4	60.18 ± 8.99	27/11 ^d^	16/1/0	7/10 ^b^	N/A	0/4/4/8/1
					Control	50/10	65.9 ± 8.1	16/76/50	15/1/0	9/6 ^b^		0/5/2/7/1
Hiroyuki Kobayashi [26]	2018	2009–2017	Japan	RCS	IONM	25/6	66.33 ^a^	4/8/5	26/3/2	17/14/0	N/A	0/11/10/9/1
					Control	44/12	63 ± 9	12/18/0	51/2/3	30/25/1		0/28/18/9/1
Zhu Weipeng [27]	2018	2015–2017	China	RCT	IONM	26/4	59.94 ± 8.966	0/42/12	30/0/0	N/A	N/A	N/A
					Control	25/5	56.16 ± 9.78	24/13 ^d^	30/0/0			
Makoto Hikage [28]	2017	2012–2015	Japan	RCS	IONM	49/5	67.6 ± 6.6	7/25/13	46/5/3	24/19/11	21/17/8/8	0/17/13/22/2
					Control	41/13	69.47 ^a^	1/11/3	48/4/2	17/6/31	28/17/8/1	0/21/15/17/1
D. Zhong [29]	2014	2008–2009	China	RCS	IONM	45/9	64 ± 8	12/18/0	49/2/3	45/26 ^c^	20/13/15/6	0/10/12/32/0
					Control	49/12	58.93 ± 9.593	0/46/17	54/3/4	49/26 ^c^	32/8/17/4	0/18/19/24/0

Abbreviations: N/A—not available; IONM—intraoperative neuromonitoring; RCS—retrospective case–control study; PCS—prospective cohort study; RCT—randomized controlled trial; SD—standard deviation; SCC—squamous cell carcinoma; AC—adenocarcinoma; CT—chemotherapy; CRT—chemoradiotherapy. Note: ^a^: Age in median (range) ^b^: Neoadjuvant therapy status in (NO/CT&CRT) ^c^: Neoadjuvant therapy status in (CT/RT) ^d^: Tumor location in upper and middle/lower.

## Data Availability

The data for this study are based on the published literature.

## Supplementary Materials

The following supporting information can be downloaded at: https://www.mdpi.com/article/10.3390/jcm12020565/s1, Supplementary Table S1: Assessment of risk of bias in non-RCTs by Newcastle-Ottawa Quality Assessment Scale; Supplementary Table S2: Sensitivity Analysis of IONM for RLNP; Supplementary Table S3: Sensitivity Analysis of IONM for Number of mediastinal LN dissected. Supplementary Table S4: Sensitivity Analysis of IONM for Aspiration. Supplementary Table S5: Sensitivity Analysis of IONM for Pneumonia. Supplementary Table S6: Sensitivity Analysis of IONM for Chylothorax. Supplementary Table S7: Sensitivity Analysis of IONM for Anastomotic Leakage. Supplementary Table S8: Sensitivity Analysis of IONM for Total LN Dissected. Supplementary Table S9: Sensitivity Analysis of IONM for POHS. Supplementary Table S10: Sensitivity Analysis of IONM for Total Operation Time.
