# Supplementary material for: Application of Intraoperative Neuromonitoring (IONM) of the Recurrent Laryngeal Nerve during Esophagectomy: A Systematic Review and Meta-Analysis
Source: J Clin Med. 2023 Jan 10;12(2):565. doi: 10.3390/jcm12020565 (PMC9860817; doi:10.3390/jcm12020565)
Supplement: Supplementary file 1 [file jcm-12-00565-s001.zip › jcm-2060815-supplementary/Supplementary Table S1 risk of bias.pdf]

Supplementary Table S1. Assessment of risk of bias in non-RCTs by Newcastle-Ottawa Quality Assessment Scale.

study	Total Score	Representativeness of the exposed cohort	Selection of the non-exposed cohort	Ascertainment of exposure	Demonstration that outcome of interest was not present at start of study	Comparability of cohorts on the basis of the design or analysis	Assessment of outcome	Was follow-up long enough for outcomes to occur	Adequacy of follow-up of cohorts
Shuhei Komatsu	9	1	1	1	1	2	1	1	1
LuoZhao	9	1	1	1	1	2	1	1	1
Masami Yuda	9	1	1	1	1	2	1	1	1
Shigeru Takeda	9	1	1	1	1	2	1	1	1
Daisuke Fujimoto	9	1	1	1	1	2	1	1	1
Hiroyuki Kobayashi	9	1	1	1	1	2	1	1	1
Makoto Hikage	9	1	1	1	1	2	1	1	1
D. Zhong	9	1	1	1	1	2	1	1	1
Chang-Lun Huang	9	1	1	1	1	2	1	1	1

Assessment of risk of bias in RCTs by Cochrane risk-of bias tool

Study	Adequate sequence generation	Allocation concealment	Blinding of participants	Blinding of outcomes	Incomplete outcome data	Selective outcome reporting	Publication AND other bias
Zhu Weipeng	Low	Low	Unclear	Unclear	low	low	low
