# Supplementary material for: Application of Intraoperative Neuromonitoring (IONM) of the Recurrent Laryngeal Nerve during Esophagectomy: A Systematic Review and Meta-Analysis
Source: J Clin Med. 2023 Jan 10;12(2):565. doi: 10.3390/jcm12020565 (PMC9860817; doi:10.3390/jcm12020565)
Supplement: Supplementary file 1 [file jcm-12-00565-s001.zip › jcm-2060815-supplementary/Supplementary Table S10 Total Operation Time.pdf]

Supplementary Table S10. Sensitivity Analysis of IONM for Total Operation Time.

Study	OR	95% CL	I2
Omitting LuoZhao	-20.34	-29.84, -10.84	0%
Omitting Masami Yuda	-9.32	-33.72, 15.07	77%
Omitting D. Zhong	-7.32	-50.63, 35.98	40%

After omitting LuoZhao's study, the pool analysis showed a decreased total operation time.

Abbreviation: IONM: Intraoperative Neuromonitoring.
