# Supplementary material for: Application of Intraoperative Neuromonitoring (IONM) of the Recurrent Laryngeal Nerve during Esophagectomy: A Systematic Review and Meta-Analysis
Source: J Clin Med. 2023 Jan 10;12(2):565. doi: 10.3390/jcm12020565 (PMC9860817; doi:10.3390/jcm12020565)
Supplement: Supplementary file 1 [file jcm-12-00565-s001.zip › jcm-2060815-supplementary/Supplementary Table S2 RLNP.pdf]

Supplementary Table S2. Sensitivity Analysis of IONM for RLNP.

Study	OR	95% CL	I^2
Omitting Shuhei Komatsu	0.38	0.27, 0.54	42%
Omitting LuoZhao	0.37	0.26, 0.53	48%
Omitting Masami Yuda	0.37	0.25, 0.54	48%
Omitting Shigeru Takeda	0.36	0.25, 0.52	48%
Omitting Daisuke Fujimoto	0.38	0.27, 0.55	41%
Omitting Hiroyuki Kobayashi	0.38	0.27, 0.55	46%
Omitting Zhu Weipeng	0.38	0.27, 0.54	45%
Omitting Makoto Hikage	0.27	0.18, 0.40	0%
Omitting D. Zhong	0.40	0.28, 0.56	47%
Omitting Chang-Lun Huang	0.38	0.27, 0.54	44%

After omitting any of the included studies, the results of pooled analysis remained robust.

Abbreviation: IONM: Intraoperative Neuromonitoring; RLNP: Recurrent Laryngeal Nerve Palsy.
