# Supplementary material for: Application of Intraoperative Neuromonitoring (IONM) of the Recurrent Laryngeal Nerve during Esophagectomy: A Systematic Review and Meta-Analysis
Source: J Clin Med. 2023 Jan 10;12(2):565. doi: 10.3390/jcm12020565 (PMC9860817; doi:10.3390/jcm12020565)
Supplement: Supplementary file 1 [file jcm-12-00565-s001.zip › jcm-2060815-supplementary/Supplementary Table S3 Number of mediastinal LN dissected.pdf]

Supplementary Table S3. Sensitivity Analysis of IONM for Number of mediastinal LN dissected.

Study	OR	95% CL	I2
Omitting LuoZhao	4.11	-0.70, 8.92	69%
Omitting D. Zhong	2.93	0.29, 5.58	0%
Omitting Chang-Lun Huang	5.13	2.77, 7.48	35%

After omitting Luo Zhao's study, the results of pooled analysis were not statistically significantly different.

Abbreviation: IONM: Intraoperative neuromonitoring.
