# Supplementary material for: Application of Intraoperative Neuromonitoring (IONM) of the Recurrent Laryngeal Nerve during Esophagectomy: A Systematic Review and Meta-Analysis
Source: J Clin Med. 2023 Jan 10;12(2):565. doi: 10.3390/jcm12020565 (PMC9860817; doi:10.3390/jcm12020565)
Supplement: Supplementary file 1 [file jcm-12-00565-s001.zip › jcm-2060815-supplementary/Supplementary Table S4 Aspiration.pdf]

Supplementary Table S4. Sensitivity Analysis of IONM for Aspiration.

Study	OR	95% CL	I^2
Omitting Daisuke Fujimoto	0.16	0.05, 0.53	0%
Omitting Hiroyuki Kobayashi	0.59	0.05, 6.39	83%
Omitting Makoto Hikage	0.60	0.06, 6.00	86%

After omitting a Makoto Hikage's study, the pooled analysis showed a decreased incidence of Aspiration.

Abbreviation: IONM: Intraoperative Neuromonitoring.
