# Supplementary material for: Application of Intraoperative Neuromonitoring (IONM) of the Recurrent Laryngeal Nerve during Esophagectomy: A Systematic Review and Meta-Analysis
Source: J Clin Med. 2023 Jan 10;12(2):565. doi: 10.3390/jcm12020565 (PMC9860817; doi:10.3390/jcm12020565)
Supplement: Supplementary file 1 [file jcm-12-00565-s001.zip › jcm-2060815-supplementary/Supplementary Table S5 Pneumonia.pdf]

Supplementary Table S5. Sensitivity Analysis of IONM for Pneumonia.

Study	OR	95% CL	I2
Omitting Shuhei Komatsu	0.59	0.42, 0.85	52%
Omitting LuoZhao	0.62	0.43, 0.90	51%
Omitting Masami Yuda	0.57	0.38, 0.84	55%
Omitting Shigeru Takeda	0.51	0.35, 0.75	44%
Omitting Daisuke Fujimoto	0.58	0.41, 0.83	55%
Omitting Hiroyuki Kobayashi	0.56	0.39, 0.80	54%
Omitting Makoto Hikage	0.48	0.33, 0.70	16%
Omitting D. Zhong	0.65	0.45, 0.95	46%
Omitting Chang-Lun Huang	0.63	0.44, 0.91	47%

After omitting any of the included studies, the results of pooled analysis remained robust.

Abbreviation: IONM: Intraoperative Neuromonitoring.
