# Supplementary material for: Application of Intraoperative Neuromonitoring (IONM) of the Recurrent Laryngeal Nerve during Esophagectomy: A Systematic Review and Meta-Analysis
Source: J Clin Med. 2023 Jan 10;12(2):565. doi: 10.3390/jcm12020565 (PMC9860817; doi:10.3390/jcm12020565)
Supplement: Supplementary file 1 [file jcm-12-00565-s001.zip › jcm-2060815-supplementary/Supplementary Table S6 Chylothorax.pdf]

Supplementary Table S6. Sensitivity Analysis of IONM for Chylothorax.

Study	OR	95% CL	I^2
Omitting LuoZhao	0.55	0.15, 2.06	37%
Omitting Shigeru Takeda	0.71	0.20, 2.59	24%
Omitting D. Zhong	0.82	0.22, 3.13	14%
Omitting Chang-Lun Huang	0.27	0.06, 1.26	0%

After omitting any of the included studies, the results of pooled analysis remained robust.

Abbreviation: IONM: Intraoperative Neuromonitoring.
