# Supplementary material for: Application of Intraoperative Neuromonitoring (IONM) of the Recurrent Laryngeal Nerve during Esophagectomy: A Systematic Review and Meta-Analysis
Source: J Clin Med. 2023 Jan 10;12(2):565. doi: 10.3390/jcm12020565 (PMC9860817; doi:10.3390/jcm12020565)
Supplement: Supplementary file 1 [file jcm-12-00565-s001.zip › jcm-2060815-supplementary/Supplementary Table S7 Anastomotic Leakage.pdf]

Supplementary Table S7. Sensitivity Analysis of IONM for Anastomotic Leakage.

Study	OR	95% CL	I2
Omitting LuoZhao	0.76	0.44, 1.31	0%
Omitting Masami Yuda	0.87	0.51, 1.49	0%
Omitting Shigeru Takeda	0.71	0.41, 1.22	0%
Omitting Hiroyuki Kobayashi	0.84	0.48, 1.47	0%
Omitting D. Zhon	0.82	0.50, 1.35	0%
Omitting Chang-Lun Huang	0.71	0.42, 1.19	0%

After omitting any of the included studies, the results of pooled analysis remained robust.

Abbreviation: IONM: Intraoperative Neuromonitoring.
