# Supplementary material for: Application of Intraoperative Neuromonitoring (IONM) of the Recurrent Laryngeal Nerve during Esophagectomy: A Systematic Review and Meta-Analysis
Source: J Clin Med. 2023 Jan 10;12(2):565. doi: 10.3390/jcm12020565 (PMC9860817; doi:10.3390/jcm12020565)
Supplement: Supplementary file 1 [file jcm-12-00565-s001.zip › jcm-2060815-supplementary/Supplementary Table S8 Total LN Dissected..pdf]

Supplementary Table S8. Sensitivity Analysis of IONM for Total LN Dissected.

Study	OR	95% CL	I2
Omitting Masami Yuda	3.81	-1.57, 9.19	66%
Omitting D. Zhong	5.72	-5.61, 17.05	79%
Omitting Chang-Lun Huang	7.52	2.44, 12.59	38%

After omitting any of the included studies, the results of pooled analysis remained robust.

Abbreviation: IONM: Intraoperative Neuromonitoring.
