# Supplementary material for: Application of Intraoperative Neuromonitoring (IONM) of the Recurrent Laryngeal Nerve during Esophagectomy: A Systematic Review and Meta-Analysis
Source: J Clin Med. 2023 Jan 10;12(2):565. doi: 10.3390/jcm12020565 (PMC9860817; doi:10.3390/jcm12020565)
Supplement: Supplementary file 1 [file jcm-12-00565-s001.zip › jcm-2060815-supplementary/Supplementary Table S9 POHS.pdf]

Supplementary Table S9. Sensitivity Analysis of IONM for POHS.

Study	OR	95% CL	I2
Omitting Masami Yuda	-1.21	-5.96, 3.54	69%
Omitting D. Zhong	-2.11	-11.61, 7.39	58%
Omitting Chang-Lun Huang	-3.96	-7.35, -0.57	0%

After omitting Chang-Lun Huang's study, the pool analysis showed a decreased POHS

Abbreviation: IONM: Intraoperative Neuromonitoring; POHS: Postoperative Hospital Stay
